# High efficiency closed-system gene transfer using automated spinoculation

**DOI:** 10.1186/s12967-021-03126-4

**Published:** 2021-11-24

**Authors:** Victoria Ann Remley, Jianjian Jin, Sarmila Sarkar, Larry Moses, Michaela Prochazkova, Yihua Cai, Lipei Shao, Hui Liu, Tatyana Fuksenko, Ping Jin, David F. Stroncek, Steven L. Highfill

**Affiliations:** 1 Center for Cellular Engineering, Department of Transfusion Medicine, NIH Clinical Center, Bethesda, USA; 2 Center for Cellular Engineering, Clinical Center, NIH, 10 Center Drive-MSC-1184, Building 10, Room 3C720, Bethesda, MD 20892-1184 USA

**Keywords:** Sepax, Spinoculation, CAR T-cell, Gene transfer

## Abstract

**Background:**

Gene transfer is an important tool for cellular therapies. Lentiviral vectors are most effectively transferred into lymphocytes or hematopoietic progenitor cells using spinoculation. To enable cGMP (current Good Manufacturing Practice)-compliant cell therapy production, we developed and compared a closed-system spinoculation method that uses cell culture bags, and an automated closed system spinoculation method to decrease technician hands on time and reduce the likelihood for microbial contamination.

**Methods:**

Sepax spinoculation, bag spinoculation, and static bag transduction without spinoculation were compared for lentiviral gene transfer in lymphocytes collected by apheresis. The lymphocytes were transduced once and cultured for 9 days. The lentiviral vectors tested encoded a CD19/CD22 Bispecific Chimeric Antigen Receptor (CAR), a FGFR4-CAR, or a CD22-CAR. Sepax spinoculation times were evaluated by testing against bag spinoculation and static transduction to optimize the Sepax spin time. The Sepax spinoculation was then used to test the transduction of different CAR vectors. The performance of the process using healthy donor and a patient sample was evaluated. Functional assessment was performed of the CD19/22 and CD22 CAR T-cells using killing assays against the NALM6 tumor cell line and cytokine secretion analysis. Finally, gene expression of the transduced T-cells was examined to determine if there were any major changes that may have occurred as a result of the spinoculation process.

**Results:**

The process of spinoculation lead to significant enhancement in gene transfer. Sepax spinoculation using a 1-h spin time showed comparable transduction efficiency to the bag spinoculation, and much greater than the static bag transduction method (83.4%, 72.8%, 35.7% n = 3). The performance of three different methods were consistent for all lentiviral vectors tested and no significant difference was observed when using starting cells from healthy donor versus a patient sample. Sepax spinoculation does not affect the function of the CAR T-cells against tumor cells, as these cells appeared to kill target cells equally well. Spinoculation also does not appear to affect gene expression patterns that are necessary for imparting function on the cell.

**Conclusions:**

Closed system-bag spinoculation resulted in more efficient lymphocyte gene transfer than standard bag transductions without spinoculation. This method is effective for both retroviral and lentiviral vector gene transfer in lymphocytes and may be a feasible approach for gene transfer into other cell types including hematopoietic and myeloid progenitors. Sepax spinoculation further improved upon the process by offering an automated, closed system approach that significantly decreased hands-on time while also decreasing the risk of culture bag tears and microbial contamination.

**Supplementary Information:**

The online version contains supplementary material available at 10.1186/s12967-021-03126-4.

## Background

Gene transfer, utilizing viral vector transduction, plays a critical role in the effectiveness of today’s cellular therapy products [1, 2]. With the FDA-approval of the first chimeric antigen receptor (CAR), Kymriah, and shortly after, Yescarta (both in 2017), there has been a flurry of other genetically modified T-cell products either waiting for approval or recently approved (such as Tecartus, approved in 2020; Abecma, approved in 2021). Gene transfer has thus become an important part of adoptive cellular therapies used to treat cancer [3–9]. For these applications, autologous lymphocytes are collected by apheresis and transduced with retroviral or lentiviral vectors containing CARs or high affinity T-cell receptors (TCR) that are specific for antigens expressed by the tumor. The transduced cells are expanded in culture for 1–3 weeks and are then infused back into the patient [10, 11]. Such an approach harnesses the power of the immune system to recognize and destroy cancer cells and has had considerable success.

Clinical cellular therapies using gene corrected lymphocytes or even corrected CD34 + cells are most effective when the transduction efficiency is high. Low transduction efficiencies are problematic since greater quantities of starting material or more prolonged post-transduction expansion would be required, significantly increasing the cost of this type of therapy. Transducing a greater proportion of cells results in a greater yield of transduced cells but due to the autologous nature of most of the current products, a large quantity of starting material is generally not possible. Patients who have been heavily treated with chemotherapy or patients with a high burden of disease, can sometimes have very few circulating lymphocytes available for collection [12, 13]. In addition to the cost of possibly extending the culture to achieve a sufficient number of cells to reach dose level, there is also the cost of using a higher multiplicity of infection (MOI) of viral vector to obtain sufficient transduction. GMP-grade viral vector production for clinical application is extremely costly, and utilizing methods that may allow for a reduction of this critical material would significantly reduce overall cost of the therapy.

The effectiveness of lentiviral- and retroviral-mediated gene transfer has been shown to be significantly enhanced when a method called spinoculation is used [14–22]. During the process of spinoculation, both the viral vector and the cells are centrifuged together to aid viral binding to the target cells. Spinoculation is routinely performed in pre-clinical laboratories using an open 6-well plate system. The virus-coated plates and the cell suspension are centrifuged together. Although this series of manipulations increases transduction efficiency, it suffers from the limitation that it is an open-system and subject to microbial contamination. Generally, this risk is offset in the preclinical laboratory by the addition of antibiotics to the culture, but this is not an option for clinical cell manufacturing. This led to developing a closed-system method to perform spinoculation gene transfer of peripheral blood lymphocytes using cell culture bags, which has been proven to increase transduction with a relatively low risk of contamination (Additional file 1: Fig. S1) [23].

While CAR T-cell production has been successful in culture bags through spinoculation, there are still limitations to this approach that we at the NIH have observed that raise challenges. If performing a large-scale transduction, multiple bags need to be prepared and possibly multiple centrifuge runs will need to be performed in order to transduce a sufficient number of cells for the designated dose level. In addition, there have been a few instances where centrifugation of a culture bag would result in leakage due to tiny tears in the bag. This leads to a potential contamination event and a deviation from the process, and the patient product is deemed not suitable for further culture or infusion. Although these events were very rare, we have nonetheless developed a spinoculation method by using an automated, closed Sepax-C Pro. The Sepax uses high speed centrifugation in a closed chamber to transduce T-cells, and then uses a pneumatic force to release the cells from the chamber into a cell culture bag to be transferred to an incubator and incubated at 37 °C. Sepax spinoculation can transduce a high number of cells using a single kit, thus avoiding processing using multiple bags in a centrifuge. The spinoculation protocol is very flexible, and allows for user adjustment of volume, cell concentration, duration of spin, and g-force at every step. The process has proven to decrease technician *hands-on* time, decrease the overall processing time, decrease the amount of viral vector required, while also decreasing the risk of possible microbial contamination.

**Fig. 1:**
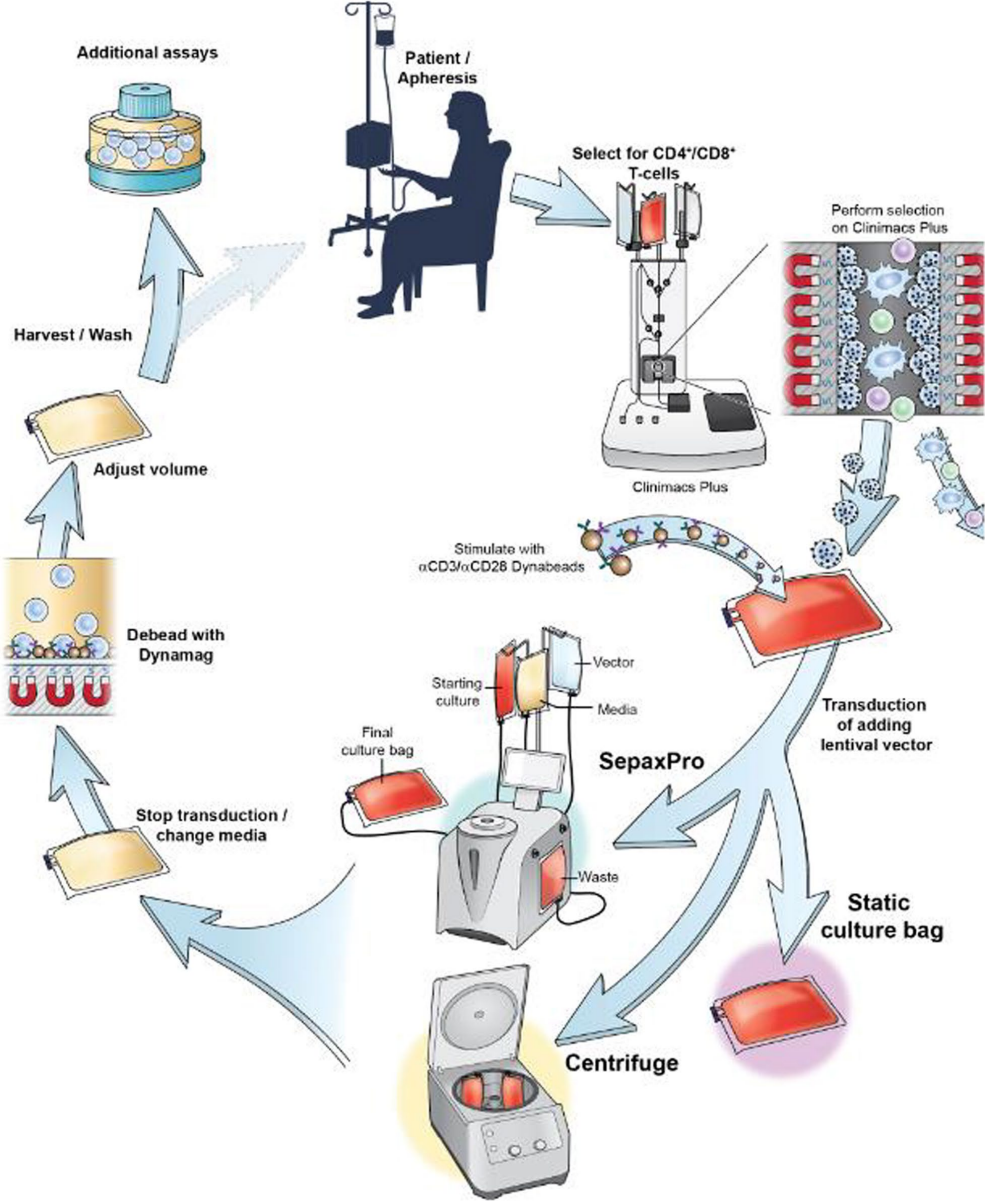
Schema of the Sepax spinoculation manufacturing process. Once the apheresis product is collected it was loaded on the Clinimacs Plus to be selected for CD4 + /CD8 + T-cells (simultaneous double positive selection). On day 0, the selected cells were incubated with anti-CD3/CD28 Dynabeads at a ratio of 3:1 (beads:cell) with a 40 IU of IL-2. These cells are incubated for 48 h before transduction. On day 2, the cells are divided into three different groups for the transduction process. For the static transduction, cells and vector were added to culture bags (Origen). Sepax spinoculation involves the cells, media, and vector be loaded to separate transfer packs and culture bags. The instrument and kit loads and washes the cells before the vector is added. The cells and vector are spun at a speed of 1000×*g* for 1 h. After spinoculation, cells are loaded into a culture bag (Origen). The bag spinoculation method is similar to the Sepax method, but the cells and vector are loaded into a culture bag (Origen), placed in an overwrap bag, and then loaded into the centrifuge buckets where they are spun at 1000×*g* for 2 h. In all methods the cells are expanded until day 9. On day 4, the Dynabeads are removed and discarded using a DynaMag CTS. On day 9 the cells are washed, assayed and cryopreserved. Cells may be thawed for functional testing, or if a clinical product, prepared for infusion in patients

We tested the Sepax to perform a fully closed spinoculation gene transfer of peripheral blood lymphocytes, and compare the performance of bag spinoculation and static bag transduction (Fig. 1). The different spin times of the Sepax were evaluated, and the optimal time was determined. We also tested different lentiviral vectors, which included a CD19/22 Bispecific CAR, a FGFR4 CAR, and a CD22 CAR. The functions of transduced cells from the Sepax were evaluated using tumor killing assays and cytokine secretion assays. Lastly, we examined gene expression patterns of the products of these processes to determine if the method of preparation influenced gene expression. The Sepax spinoculation transduction was found to be comparable to the bag spinoculation while not affecting the function of the CAR T-cells.

## Materials and methods

### Cells and viral vector

PBMC concentrates were collected by apheresis (Optia, Fenwal or Spectra, Terumo BCT), and CD4^+^ and CD8^+^ lymphocytes were selected by CliniMACS (Miltenyi). Three different lentiviral vectors were used for the CAR T-cell experiments. The CD19/22 Bispecific CAR (Lentigen Technology, Gaithersburg, MD) was used for optimization experiments and additional CAR experiments at a MOI of 10. The FGFR4 CAR (Lentigen Technology, Gaithersburg, MD) was used in one experiment at a MOI of 10. The CD22 CAR (Lentigen Technology, Gaithersburg, MD) was used for one experiment at a MOI of 2.

### Cell culture and lentiviral vector transduction

Cryopreserved CD4/CD8 selected lymphocytes either from healthy donor or patient were thawed on day 0, and cultured at 2 × 10^6^/mL and activated for 48 h in AIM-V media (Gibco, Grand Island, NY) containing 5% heat inactivated human AB serum (Valley Biomedical, Winchester, VA), 2 mM Glutamax (Gibco), 40 IU/mL IL2 (Prometheus Laboratory, Inc. San Diego, CA) and stimulated with CD3/CD28 Dynabeads (Invitrogen, Camarillo, Ca) with a ratio of 1 cell: 3 beads. On day 2, cells were transduced with CD19/22 Bispecific CAR, FGFR4 CAR or CD22 CAR lentiviral vectors (Lentigen Technology, Gaithersburg, MD) either using bag spinoculation or Sepax spinoculation. Static bag transduction and non-transduced (no vector added) culture were used as controls. A MOI of 10 was used for CD19/22- and FGFR4-CAR vectors, while a MOI of 2 was used for the CD22-CAR vector. For the bag spinoculation group, cells and vectors were added into PL-30 culture bags and spun at 1000×*g* for 2 h at 32 °C. Once the spin was complete, the buckets were removed carefully and the bags, which were sealed in overwrap bags, were removed before transfer to the incubator. For the Sepax spinoculation group, the bags containing cells and vectors were sterilely connected to the Sepax kit, and spinoculation was performed automatically as described in the following section (Cytiva Sepax for Spinoculation). To evaluate the optimal spin time, 0.5 h, 1 h and 2 h were tested. After spinoculation was completed, cells were transferred into PL bags (OriGen Biomedical, Austin, TX), and placed in the incubator. Media change occurred 24 h after transduction and de-beading occurred on day 4 of the culture before being diluted to 0.4 × 10^6^/mL. The cells were diluted on day 7 to 0.6 × 10^6^/mL and harvested on day 9. Fold expansion was calculated from post de-beading (day 4) to day 9. Cells were cryopreserved at the end of the culture using Cryostor-CS10 (Biolife Solutions) for future in vitro functional studies.

### Cytiva sepax for spinoculation

CD4/CD8 Selected T-cells were transferred to a 150 mL transfer pack, 350 mL AIM-V 40 CM was transferred to a 600 mL transfer pack, 12 mL AIM-V 40 CM containing the vector and protamine sulfate (0.3 mL of 1 mg/mL) was transferred to a PL30 culture bag sealed in half. 3-5 mL of air needed to be added to the bag containing the vector. 1.2 × more vector for the specific MOI needed to be prepared in the 12 mL of media to have enough to prevent loss of vector. The Sepax instrument pulled in 10 mL in order to use the correct amount of vector for the MOI calculated. A Sepax-C Pro Kit Reference CT-60.1 kit was used and the bags containing reagents were sterile connected. The tubing for the vector needed to be as short as possible to avoid losing vector during addition. The Washing Application-Spinoculation was selected and once all the parameters are set, the kit was loaded. The Sepax pulled the cells through tubing in the machine into a chamber where they were washed and pelleted before the vector was added. Once the instrument brought the vector in through the tubing, the cells and vector were spun for the programed time. When the transduction was finished the cells were pushed back through the tubing into a sterile culture bag. The chamber was rinsed and the media was added to the culture bag with the cells. After the process stopped, the cells were transferred to the culture bag and the chamber was rinsed with enough media to bring the cells to a concentration of 0.5 × 10^6^/mL of the starting viable total nucleated cells (TNC).

### In vitro cytokine and NALM6 tumor clearance assays

Tumor killing assays were performed with CD19/22 and CD22 CAR T-cells in order to test the downstream effects of Sepax spinoculation on the function of the cells. 5 × 10^4^ or 25 × 10^4^ CAR T-cells were incubated with NALM6 (B cell precursor leukemia) tumor cells in either a 1:1 or 5:1 (CAR T-cell: NALM6) ratio in 96 well plates. Cells were cultured in RPMI (Lonza, Walkersville, MD) + 10% FBS (HyClone Laboratories, Logan, Utah) for both 24 h and 48 h at 37 °C. T-cells for these experiments were all thawed, cultured, and harvested the same way for all three experiments. The T-cells were resuspended to 25 × 10^5^/mL based on the harvest transduction efficiency. Groups included bag centrifuge transduced CAR T-cells at effector to target ratios of 1:1 and 5:1, Sepax transduced CAR T-cells at ratios of 1:1 and 5:1, Static transduced CAR T-cells at ratios of 1:1 and 5:1, untransduced control T-cells at ratios of 1:1 and 5:1, and NALM6 only. For each condition tested, T-cells were plated in 3 wells with tumor cells in a 96 well plate for each timepoint. After 24 and 48 h of incubation, the cells were collected and stained for analysis by flow cytometry. Supernatant from the co-cultures were saved for pro-inflammatory cytokine analysis using ELISA.

### Cytokine secretion assays

Supernatant collected from the killing assays were stored at − 80 °C. ELISA kits were purchased from R&D Systems (Minneapolis, MN). TNF-α and IFNγ assays were performed according to the kit’s instructions. Only the samples incubated at an effector to target ratio of 5:1 were tested. For all of the IFNγ assays, samples were diluted 1:10 and for the TNFα assays the samples were diluted to a 1:3. Assay diluent was combined with the supernatant samples and incubated for 2 h at room temperature. A washing step of four washes occurred before the IFNγ or TNFα conjugate was added for another 2-h incubation. A second washing step occurred and then the substrate solution was added and incubated for 30 min in the dark. Stop solution was added after to stop the overall reaction. The plates were read at 450 nm and 570 nm. On each assay plate, a standard curve was made following the manufacturer’s instructions and using the given standard samples. Optical density values were analyzed using Prism and Excel. The Standard curves (R values of 0.99) were used to calculate the actual optical densities of each sample.

### Vector copy number assays

The VCN assay was performed using both commercial and unique primers to identify the number of copies of the vector integrated into each transduced cell. The vector copy number for all the transduced groups were less than five copies per transduced cell in accordance to FDA regulations. Genomic DNA was extracted from CAR T-cell samples and untransduced control cells using DNeasy Blood and Tissue Kit (Qiagen). The purity and concentration of the DNA samples were measured using a Nanodrop spectrophotometer (Thermo Fisher Scientific). The VCN assay was performed using Auto DG QX200™ ddPCR system (Bio-Rad laboratories), in brief, 40 ng genomic DNA from each sample was used for VCN assay, the generation of droplets was performed by the QX200 Auto DG, after the PCR reaction, the droplets were analyzed with QX200 Droplet Reader and QuantaSoft software version 1.7.4 (Bio-Rad) following manufacturer’s protocols.

### Gene expression

Total RNA was isolated from frozen CAR T-cell samples using an miRNeasy mini kit (QIAGEN) and quantified by 2100 Bioanalyzer (Agilent). 100 ng of total RNA was hybridized in solution with the nCounter CAR-T Characterization panel (Nanostring Technologies) at 65 °C for 17 h. The hybridized samples were loaded into the nCounter CAR-T cartridge, which was then sealed and placed in the instrument for processing. RCC files containing raw counts for 780 genes were generated and imported into nSolver Analysis Software 4.0 to normalize. Downstream analysis was performed in a Rstudio environment or Partek Genomic Suite 7.0 (Partek Inc., St. Louis, MO, USA) data visualization and hierarchical cluster analysis.

### Flow cytometry

The transduction efficiency and the proportion of cells expressing CD3, CD4 and CD8 were measured by flow cytometry (BD FACS Canto, BD Biosciences, San Jose, CA) after labeling with fluorescent labeled antibodies. The CD22-CAR vector transduction efficiency was detected using protein L. The CD19/22-CAR vector transduction efficiency was measured using biotinylated Protein L/PE-labeled Streptavidin, rhSiglec-2 (RnD), and biotin-CD19 Fc (Miltenyi). The FGFR4-CAR construct contained a truncated EGFR (tag), and was measured using anti-EGFR. The cells used in the killing assay experiments were further tested using 7AAD, CD3 and CD45. NALM6 cells expressed GFP and were identified under the FITC channel (Cytoflex).

### Graphs and statistical analysis

**Fig. 2:**
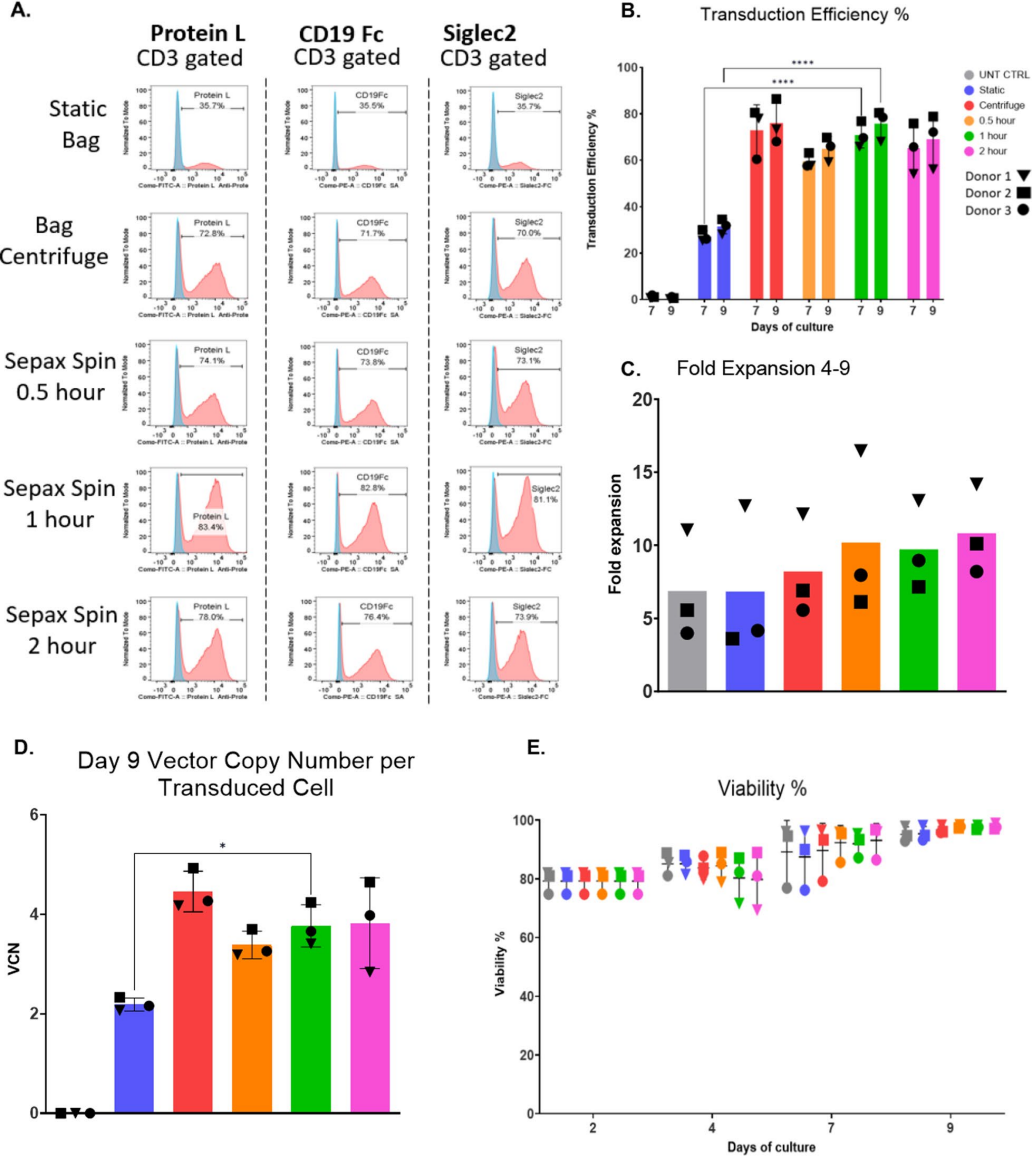
Closed system Sepax spinoculation is an alternative to bag spinoculation. T-cells were stimulated with CD3/28 dynabeads before being divided between 6 different conditions: Bag centrifuge, 2-h Sepax, 1-h Sepax, 0.5-h Sepax, Static bag, or untransduced control (UNT CTRL). **A** Representative transduction efficiency from flow histograms are shown. **B** Cumulative flow transduction efficiency is shown. **C** Fold expansion as calculated from day 4–9. **D** The vector copy number per transduced cell at day 9. **E** Viability among groups on Day 0 and Day 9, with all groups being near 100% viable on day 9. Mean and SD of triplicate experiments are shown ****Indicates p ≤ 0.0001 between the static and Sepax groups

Graphpad Prism 7 software was used to make all the graphs. A 2-way Anova was used to compare the significance between each group in Fig. 2B, Fig. 5D–F, and Additional file 1: Fig. S2C1–C3. Welch’s *t* test was used for Fig. 2D. An ordinary One-way ANOVA was used for 4B and Additional file 1: Fig. S1C, and unpaired *t* tests were used for Fig. 4D.

## Results

### Closed system bag spinoculation increases transduction efficiency for lentiviral vectors

Early experiments using a process designed for retroviral gene transfer (Additional file 1: Fig. S1A, B) showed that the effectiveness of transduction can be significantly enhanced with bag centrifuge spinoculation compared to static transduction and the results were comparable to plate spinoculation (Additional file 1: Fig. S1C). In addition, we showed that this increase in transduction due to the spinoculation did not come at the cost of lower cell expansion or viability (Additional file 1: Fig. S1D, E). Further, we investigated if there were any detrimental effects of the addition of Dynabeads during the spin process. In this experiment, 1.5 × 10^7^ cells plus 4.5 × 10^7^ Dynabeads (3:1 bead to T-cell ratio) were transferred to a PL30 bag, and the same number of cells without beads was transferred to a second bag as a control. A single 1000×*g* centrifugation was performed using cells with and without anti-CD3/CD28 beads. Cells were centrifuged for 2 h and cultured for 7 days. Cell number and viability were compared at post-centrifugation, and again at culture days 4 and 7. As shown in Additional file 1: Fig. S1F and G, cell number and viability of paired samples were nearly identical throughout the culture process, demonstrating that spinoculation with CD3/28 Dynabeads exhibited no detrimental effects on cell viability or expansion.

### Automated closed system sepax is an alternative to bag spinoculation

Although bag spinoculation increases transduction efficiency, it suffers from the limitation of the maximum cell number for each bag, thus multiple bags and multiple rounds of centrifugations are required for high dose clinical products. This not only requires more total and hands-on time but increases the chance of microbial contamination. Therefore, we decided to test spinoculation of T-cells in the Sepax-C Pro to address these issues and streamline the process. To optimize the Sepax procedure, several durations of Sepax spinoculation (2-h, 1-h, 0.5-h) were compared to bag spinoculation using a 2-h bag centrifuge time. For these studies, we utilized a lentiviral vector encoding CD19/CD22 CAR at a MOI of 10. Transduction efficiencies from Sepax prepared cells were not significantly different from the 2-h bag spinoculation for CD19/22 CAR T-cells transduction efficiency, with the Sepax spinoculation 1-h results being significantly better than the static transduction (83.4% vs. 35.7%) (Fig. 2A, B). The fold expansion throughout the culture period was comparable for all transduction methods and was similar to the untransduced control cells (Fig. 2C). Donor 1 expanded more than the other two donors for all of the groups tested. There was no significant difference between the vector copy number between all three timepoints of the Sepax spinoculation compared to the 2-h bag spinoculation cells (data not shown). The vector copy number results were similar to the transduction efficiency results, with the 1-h Sepax spinoculation CAR T-cell transduction efficiency being significantly higher than that of static transduced cells (Fig. 2D).

The viability of the CAR T-cells was consistent among all groups throughout the culture period and by day 9 the viability was close to 100% (Fig. 2E). The 1-h Sepax spinoculation condition was chosen for the remaining the experiments due to having comparable results to the 2-h Sepax and bag spinoculation conditions and needing less total time for transduction compared to the 2-h Sepax spinoculation.

### Sepax spinoculation is suitable for patient samples

**Fig. 3:**
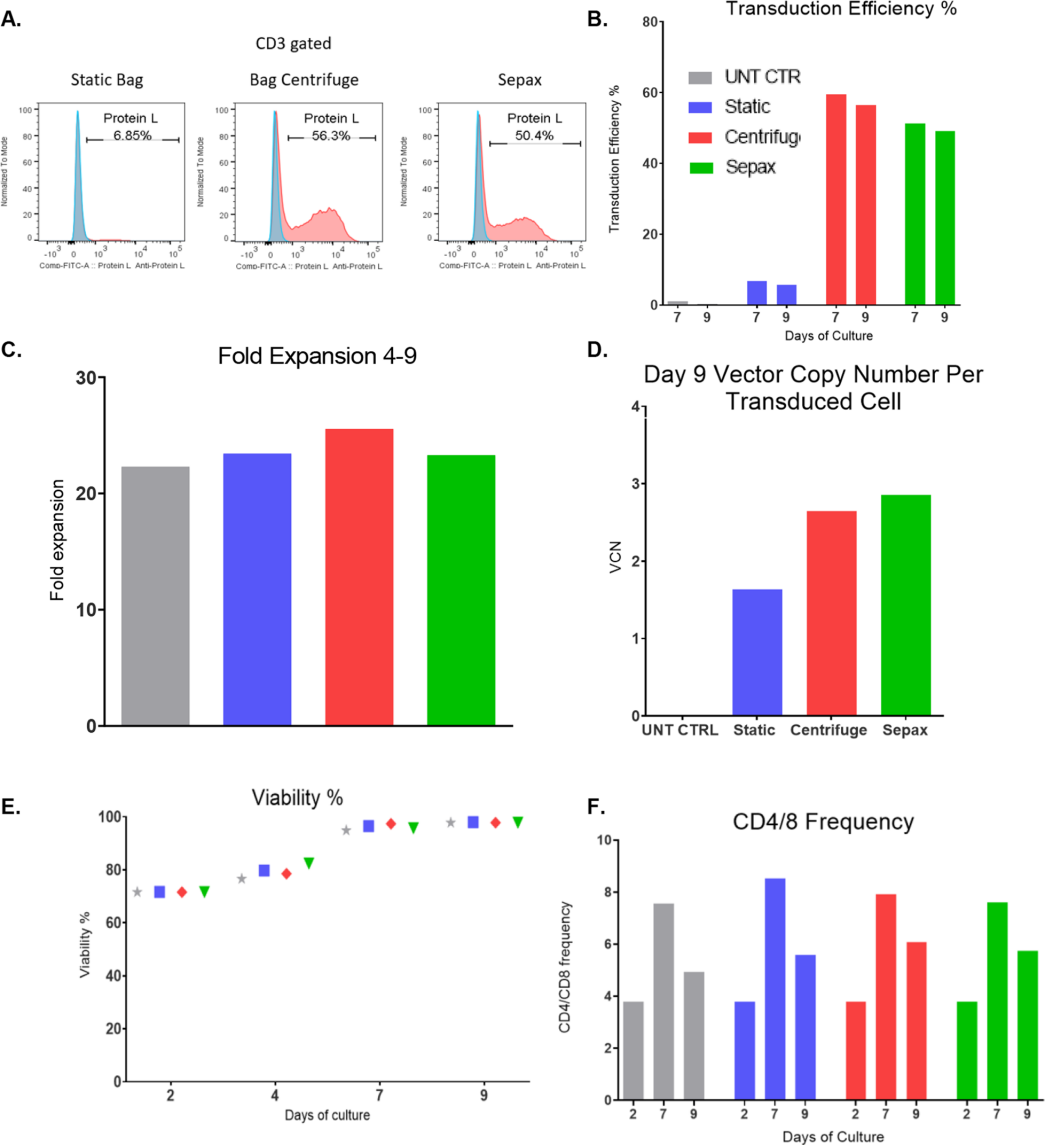
Sepax spinoculation is suitable for patient samples. T-cells were prepared in the same manner as in Fig. 2, however only a 2-h bag centrifuge, 1-h Sepax, Static bag, and UNT CTRL groups were used. Patient CD19/22 CAR T-cells were produced with all three methods. Representative transduction efficiencies (**A**) and cumulative transduction data (**B**) on day 9 is shown. **C** T-cell fold expansion from day 4–9 was calculated. **D** VCN was calculated using ddPCR. **E** Viability was tested using AOPI. **F** CD4/8 frequencies were obtained by flow cytometry and plotted

The 1-h Sepax spinoculation condition was tested against the 2-h bag spinoculation and traditional static transduced conditions for a patient sample with the CD19/22 Bispecific CAR vector. The CD4/CD8 cells were prepared and transduced in the same manner as the previous optimization experiments. When testing the patient’s cells, the 1-h Sepax spinoculation condition was comparable to the 2-h bag spinoculation condition (Sepax: 50.4%, Centrifuge: 56.3%) (Fig. 3A, B) and both had higher transduction efficiencies than static transduction. Fold expansion was comparable between all groups throughout the culture period (Fig. 3C). The patient cells expanded more than the healthy donor cells in the previous experiments. The vector copy number results for the patient sample reflected the transduction efficiencies and the vector copy number results were within the FDA limit of < 5 copies/transduced cell (Fig. 3D). Cell viability was comparable between all groups throughout the culture period (Fig. 3E). For the patient cells the CAR T-cell CD4/8 ratio was consistent between the Sepax spinoculation, bag spinoculation, and static transduction cells and the untransduced cells (Fig. 3F). These results show that it is possible to use the Sepax spinoculation method for transduction of patient samples with acute lymphoblastic leukemia (ALL), which are often described as being more difficult to transduce and expand.

### Sepax spinoculation is suitable for other lentiviral vectors

**Fig. 4:**
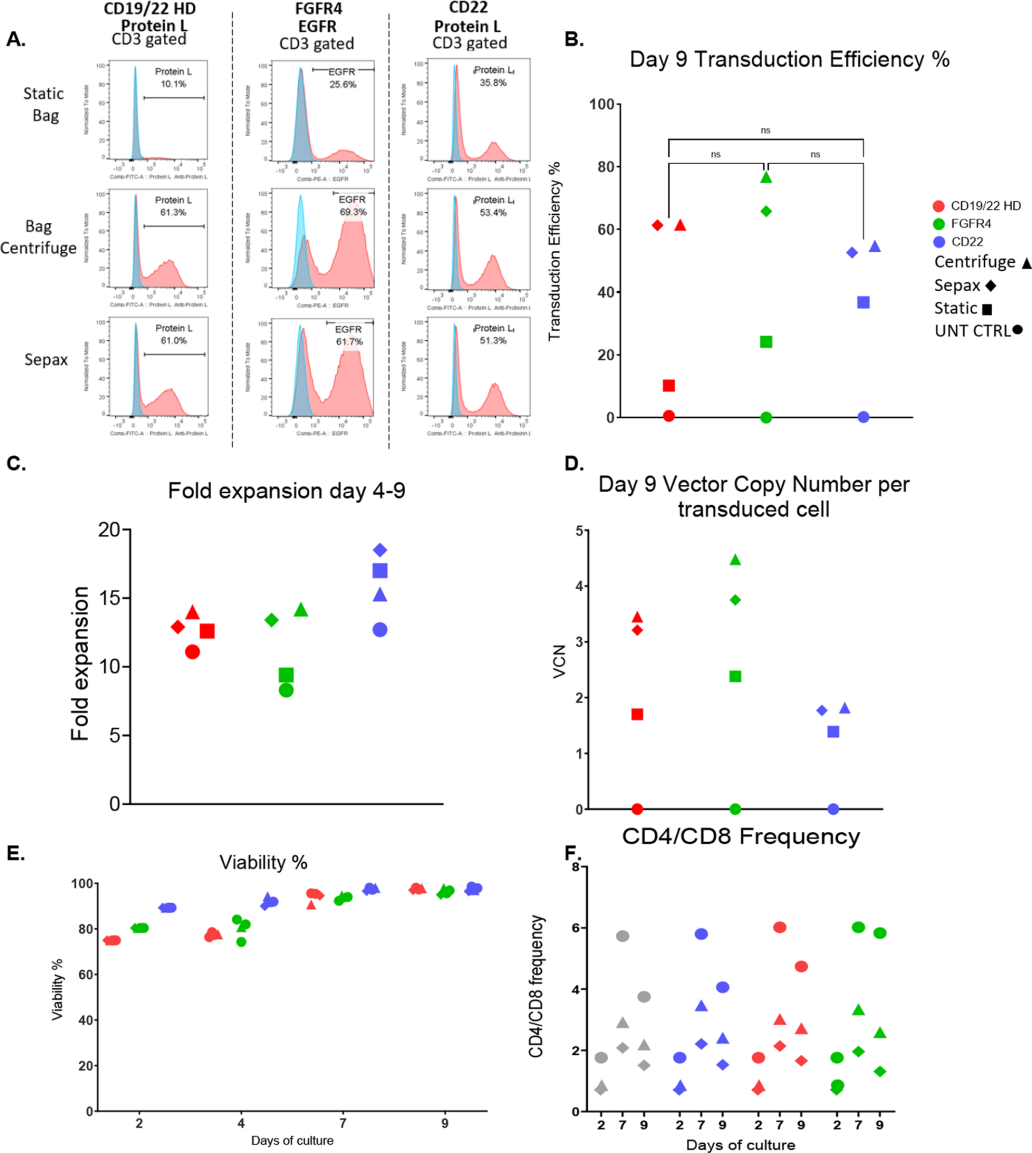
Sepax spinoculation is suitable for other lentiviral vectors. Three CAR vectors; CD19/22 Bispecific, FGFR4, and CD22 were compared to see if the Sepax spinoculation can be performed for multiple CAR productions. Representative transduction efficiencies (**A**) and cumulative transduction data (**B**) on day 9 is shown. **C** Fold expansion from day 4–9 was calculated. **D** VCN was calculated using ddPCR. **E** Viability was tested using AOPI.** F** CD4/8 frequencies were obtained by flow cytometry and plotted

Two additional CAR lentiviral vectors, FGFR4 and CD22 were also tested for transduction using the Sepax spinoculation method to ensure results observed were not dependant on the CAR construct used. For all experiments using the CD19/22- and FGFR4-CAR vectors, a MOI of 10 was used. However, when using the CD22-CAR vector, a MOI of 2 was used. The same healthy donor cells were used for the studies with the CD19/22 Bispecific and FGFR4-CAR vectors and cells from a second healthy donor was used for the CD22 CAR vector studies. The transduction efficiency for the FGFR4-, CD22-, and CD19/22-CAR vectors were comparable with no significant differences among the bag and the Sepax spinoculation groups (Fig. 4A, B). Fold expansion was consistent among all conditions for each CAR (Fig. 4C). The vector copy number correlated with the transduction efficiency for each group, and there was no significant difference between the bag and Sepax spinoculation transduction methods (Fig. 4D). The viability was consistent throughout the culture and by day 9 the viability was close to 100% among all CAR T-cells produced using Sepax, bag, and static transduction methods and the untransduced controls (Fig. 4E). The CD4/8 ratio was compared for these experiments to see if there were any changes between conditions. The ratio was consistent among the transduction groups across each CAR vector (Fig. 4F). These results show that it is possible to use the Sepax spinoculation method for transduction of multiple CAR lentiviral vectors, and the transduction enhancement is not vector specific.

### Functional characteristics of CAR T-cells are not affected by spinoculation

**Fig. 5:**
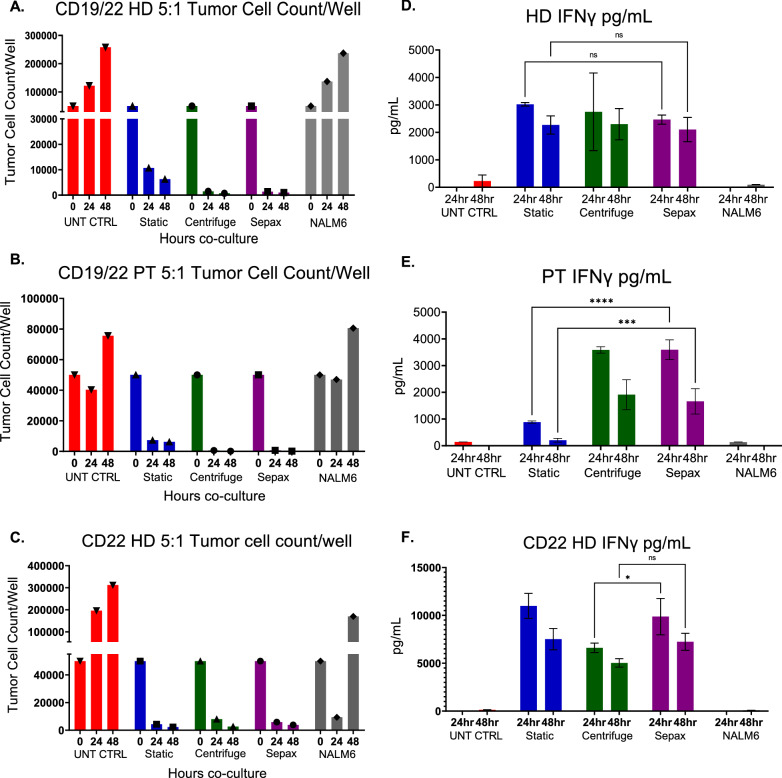
Functional characteristics of CAR T-cells are not affected by spinoculation. Cryopreserved CD19/22 CAR T-cells from a healthy donor, patient, and CD22 CAR T-cells were thawed and co-cultured with NALM6 tumor cells. 1:1 and 5:1 ratio of CAR T-cells to tumor cells were cultured together for 24 and 48 h. **A**–**C** Tumor Cell Counts per well calculated from cell counts and flow cytometry for healthy donor CD19/22 CAR T-cells (**A**), patient CD19/22 CAR T-cells (**B**), and healthy donor CD22 CAR T-cells (**C**). IFNγ ELISA’s were performed to measure the cytokine levels produced by the T cells in the supernatant for healthy donor CD19/22 CAR T-cells (**D**), patient CD19/22 CAR T-cell (**E**), and healthy donor CD22 CAR T-cells (**F**). Mean and SD of triplicate wells are shown.*Indicates p ≤ 0.05, ***Indicates p ≤ 0.001, ****Indicates p ≤ 0.0001 between the comparison of Sepax and centrifuge; and the static and Sepax groups

In order to confirm that the Sepax spinoculation did not affect the functional properties of the CAR T-cells, a killing assay was set up using NALM6 tumor cells, which expresses both CD19 and CD22 targets along with GFP that was used to quantitate the cells. CAR T-cells were cultured with the tumor cells in either a 1:1 or a 5:1 ratio for 24 h and 48 h. CD19/22 CAR T-cells derived from the patient and the healthy donor cells, along with the CD22 CAR T-cells from the additional healthy donor were used. In the CD19/22 experiments, the tumor cell counts per well decreased by approximately 75% for the static transduced CAR T-cells when tested at 5:1 while this cytotoxic effect was even greater for the CAR T-cells produced by a bag and Sepax spinoculation tested at 5:1. By 48 h there were almost no tumor cells remaining for CAR T-cells produced by bag and Sepax spinoculation tested at 5:1 (Fig. 5A, B Additional file 1: Fig. S2A, B). For the CD22 CAR T-cells, cytotoxicity between groups appeared to be more consistent. For CD22 CAR T-cells produced with each of the 3 transduced methods, target cell counts decreased by more than half by 24 h (Fig. 5C).

ELISA’s were used to detect IFNγ and TNFα from the supernatant from each of the above experiments. The CD19/22 CAR T-cells produced from healthy donor cells using each of the 3-transduction method released large quantities of IFNγ (~ 3000 pg/mL) and IFNγ released by the CD19/22 CAR T-cells produced using static transduction, bag spinoculation, and Sepax spinoculation was comparable (Fig. 5D). It is interesting to point out that the CAR T-cells produced using static transduction were observed to have less killing at the 24-h time point despite having approximately the same level of IFNγ production. The levels of TNFα released by each of these CAR T-cells were lower than the IFNγ levels. The quantities of TNFα released by CD19/22 CAR T-cells produced by bag and Sepax spinoculation were comparable and higher than the quantities released by CAR T-cells produced by static transduction at both 24 and 48 h (~ 400 pg/mL) (Additional file 1: Fig. S2C).

The cytokine release by CD19/22 CAR T-cells produced from patient cells was similar to that released by healthy donor CD19/22 CAR T-cells. For IFNγ production, the CAR T-cells manufactured by bag and Sepax spinoculation were comparable at both 24 and 48 h (~ 3500 pg/mL). However, the CAR T-cells produced by static transduction had a significant decrease in cytokine levels which correlated to the lack of killing of tumor cells (Fig. 5E). The absolute values of TNFα were again lower than IFNγ, as expected. The TNFα produced by CAR T-cells manufactured using bag spinoculation was higher than that produced Sepax spinoculation CAR T-cells at 24 h, but was comparable at 48 h. The TNFα levels dropped at 48 h for the bag and Sepax spinoculation CAR T-cells to ~ 200 pg/mL. We observed a statistically significant decrease in cytokine production by CD19/22 CAR T-cells manufactured by static transduction at both 24 h and 48 h compared to the Sepax and bag spinoculation CAR T-cells (Additional file 1: Fig. S2D).

IFNγ production by CD22 CAR T-cells was approximately equivalent for cells produced by static and the Sepax spinoculation transduction. Both cell types produced approximately 10,000 pg/mL at 24 h. All 3 types of CD22 CAR T-cells experienced a decline in IFNγ production at 48 h. At 24 h, the bag spinoculation group produced significantly less IFNγ than the two other groups, but the levels declined for all the groups at 48-h, making the differences non-significant (Fig. 5F). The TNFα levels were not different between the static transduction, bag spinoculation, and Sepax spinoculation CD22 CAR T-cells at 24 h. The TNFα levels appeared to rise in the static transduction CAR T-cells at the 48-h time point, whereas they dropped in the bag and Sepax spinoculation CAR T-cells at the same time point (avg, 1045 pg/mL, 558 pg/mL, and 343 pg/mL) (Additional file 1: Fig. S2E). We conclude that using the Sepax instrument for transduction did not affect the killing properties of the CAR T-cells, but may influence cytokine secretion at early time points after re-activation, and that the process of spinoculation in general may be beneficial for some patient samples, potentially by promoting activation and enhancing cytokine secretion.

### Gene expression is not affected by spinoculation

**Fig. 6:**
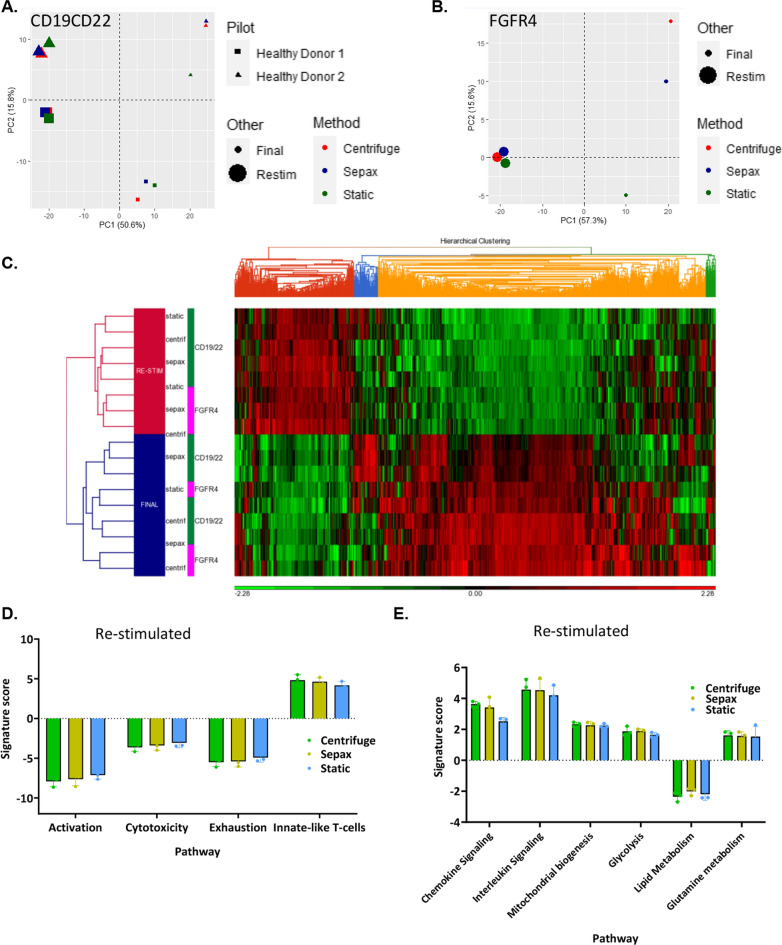
Gene expression of the final product post spinoculation vs re-stimulated CAR T cells are similar. PCA analysis PC1 vs PC2 comparing the different transduction methods and 8-day cultured samples to re-stimulated samples between two different healthy donors for CD19/22 transduction (**A**) and FGFR4 transduction (**B**). **C** Hierarchical clustering analysis of CD19/22 (**C**) and FGFR4 CAR T-cells before and after re-stimulation. Comparison of genes for Activation, Cytotoxicity, Exhaustion, ad Innate-like T cell functions were compared across each re-stimulated transduction method (**D**). Comparison of genes for Chemokine signaling, Interleukin Signaling, Mitochondrial biogenesis, Glycolysis, Lipid Metabolism, and Glutamine metabolism functions across each re-stimulated transduction method (**E**)

CAR T-cell samples from two healthy donors cryopreserved at the end of the culture were thawed and rested overnight in complete medium in a 37 °C tissue culture incubator. Some cells were used to represent the clinical CAR T-cells that would be taken out of culture and prepared for patient injection (depicted as *final*). Another set of the cultured CAR T-cells were incubated for 24 h after an initial resting period with CD3/CD28 Dynabeads to simulate what would occur during T-cell re-activation following the infusion of CAR T-cells that had not be cryopreserved. When subjected to PCA analysis the final CD19/22 CAR T-cells clustered together nicely by donor and method, with the biggest difference being the static culture from healthy donor 2 (Fig. 6A). The re-stimulated cells showed even less variability than the final cells and formed very tight clusters (Fig. 6A). These results indicate that there are no large differences in gene expression that is a result of the transduction method.

PCA analysis of the FGFR4 CAR T-cells displayed a difference in final cultured cells, again with the bag and Sepax spinoculation group clustering separate from the static transduced CAR T-cells (Fig. 6B). Similar to the CD19/22 CAR, upon re-stimulation, there was much less variability between these groups (Fig. 6B).

The results of unsupervised hierarchical clustering analysis of the CD19/22 CAR T-cells and the FGFR4 CAR T-cells (Fig. 6C) were similar to the PCA analysis. Cells that were re-stimulated clustered separately from those that were not. When examining specific pathways important for T-cells, there was no observable difference between the CAR T-cells produced using the three different methods (Bag, Sepax, and Static) (Fig. 6D, E).

### Discussion

The work described highlights the use of the automated closed system gene transfer system for clinical scale manufacturing of various CAR T-cells. Conventional methods typically use open 6-well plates or culture bags to perform spinoculation for the manufacture of clinical cell therapies. We tested an automated Sepax-II Pro instrument for spinoculation gene transfer of peripheral blood lymphocytes. The closed-system Sepax spinoculation described here reduces the risk of microbial contamination of cell preparations and reduces the risk of viral vector spread within the cell therapy facility clean room. Total time of the transduction process and technician *hands-on* time is also decreased due to having the ability to perform one large scale run compared to having to implement multiple centrifuge cycles for a patient. Sepax spinoculation more readily lends itself to clinical large-scale transduction and automation than the static or bag/plate centrifuge transduction, and is effective for both retroviral and lentiviral vectors. This new method was able to generate a large quantity of functional CAR T-cells that were capable of recognizing and killing tumor cells in culture. The closed system method is reproducible for various CAR lentiviral vectors and is shown here to be applicable to patient samples with little variation from healthy donor samples.

Besides the Sepax, there are other commercially available devices that are emerging that may be able to enhance transduction efficiency using a spinoculation technique. The Miltenyi Prodigy instrument, for example, is another closed system transduction system that is currently beta testing a process for the same purpose. The spin duration, speed (up to 400×*g*), and temperature (+ 4 to + 38 °C) can be programed by the technician, which may be useful for development purposes. The Sepax is similar in that each step in the transduction protocol is modifiable, and therefore, each step can be fine-tuned to ensure optimal transduction occurs. Sepax spinoculation also allows for larger volumes, spin times, and spin speeds from 500 to 1200×*g*. One caveat to the Sepax instrument is that it currently only operates at room temperature. Although, this appears to be sufficient to enhance transduction efficiency, literature in the field would suggest increasing the temperature to 32 °C may result in even more dramatic effects [24, 25].

The study described here used a single 1-h Sepax program, transducing 60 × 10^6^ T-cells, but this number can be doubled to 120 × 10^6^ T-cells for larger patient transductions. The transduction process was designed to be incorporated at clinical cell processing facilities and utilized standard equipment that are commonly available at these sites. First, CD4 + and CD8 + T-cells were selected by the automated CliniMACS (Miltenyi). Once the T-cells were activated with CD3 + /CD28 + Dynabeads (Invitrogen, Camarillo, Ca), they were transduced using either the Sepax spinoculation, bag spinoculation, or remained static during the transduction. Cells were fed and expanded over a period of 7 days after transduction in culture bags. Once the cells are harvested they are washed with plasmaLyte-A containing HSA and resuspended at a final concentration on 10 × 10^6^ cells/mL. These cells were then assayed for vector copy number, flow cytometry, and frozen down for future functional assays.

We confirmed that this process results in very consistent products, capable of recognizing and killing tumor cells. We did however experience some differences in cell growth and transduction efficiency based on different donors and different CAR lentiviral vectors. The patient samples expanded better than the healthy donors, but some healthy donors had better transduction efficiency than the patient. Variability between patient samples, and even between different healthy donor samples is commonly observed. Therefore, we tried to keep our focus on differences between the three groups (static, bag centrifuge, Sepax) within a particular healthy donor or patient sample.

When comparing multiple Sepax spin times against the 2-h bag centrifuge and static transduction, we found that we were able to use a Sepax spin time of 1-h that was comparable to the two-hour centrifuge approach, eliminating at least an entire hour from the total processing time (Fig. 2). If only one centrifuge is available within a tissue culture room and > 4 spins are required, this could potentially result in saving several hours rather than only one. The 1-h Sepax spinoculation was then tested against the bag centrifuge and static transduction using a sample from a patient with B-cell-ALL. Transduction efficiency was similar between the Sepax and bag centrifuge spinoculation groups (Fig. 3A, B), and both of these were significantly higher than static transduction (~ 10% TE static vs. ~ 55% for centrifuge and Sepax). This highlights the power of using the spinoculation approach for patient samples that tend to be more difficult to transduce. The 1-h Sepax spinoculation was also tested for the FGFR4 CAR (Lentigen Technology, Gaithersburg, MD) and CD22 CAR vector (Lentigen Technology, Gaithersburg, MD). The transduction efficiencies were comparable among the different CAR vectors for the Sepax spinoculation groups (Fig. 4A, B). It is important to note that the MOI used for the CD22 CAR vector was reduced to MOI = 2 for the studies presented here. Initial experiments using a MOI = 10 resulted in very little difference between static and spin approaches (data not shown). We speculate that the vector was saturating under these circumstances and required dilution to observe effects on transduction. The obvious positive attribute is that we are able to obtain high transduction with the Sepax spinoculation approach when using significantly less vector, resulting in a substantial cost-savings since GMP vector production tends to be very expensive.

Functional tumor cell killing assays and cytokine release assays showed that the Sepax spinoculation transduction method did not affect the tumor recognition and killing functions of the CAR T-cells (Fig. 5). The main differences between the CAR T-cells produced with the different CAR lentiviral vectors were due to differences in vector constructs, rather than differences in the cell manufacturing process. Nanostring gene expression comparison also helped conclude that the Sepax spinoculation had very little effect on the functional properties of CAR T-cells, and major pathways utilized in the T-cells ability to kill tumor cells appeared unaffected (Fig. 6).

Concurrent with this study, optimization of the Sepax spinoculation method for gamma-retroviruses is in the process of being developed. Testing to determine if the Sepax spin chamber can be coated with material commonly used to enhance transduction is underway.

## Conclusion

We developed an automated closed-system spinoculation gene transfer method that is less prone to contamination than the conventional open plate method and bag spinoculation, thereby improving upon the current manufacturing procedures for CAR T-cells. This method is in the process of being implemented here at the NIH Clinical Center for upcoming clinical trials. This manufacturing process allows for consistent final products with no observed negative attributes on the function of the cells. This approach allows for safer and easier manufacturing process, which can benefit cell therapy manufacturing centers.

## Supplementary Information

**Additional file 1: Figure S1. T-Cell Retroviral gene transfer is enhanced using a closed-system spinoculation with CD3/CD28 Dynabeads**. Schema of culture (A1) and image of centrifuge bucket orientation (B). Lymphocytes were stimulated with rIL-2 and OKT3. After 2 days in culture cell were transduced with E6 TCR retroviral vector in 6 well plates using spinoculation or bags with and without spinoculation. The transduction process was repeated on day 3. After 7 days of culture the cells were evaluated for transduction efficiency (C), fold T cell expansion (D), and cell viability (E). PBMCs enriched for lymphocyte by density gradient centrifugation were placed in PL30 bags with rIL3 and OKT3 antibody with and without CD3/CD28 Dynabeads. To simulate spinoculation, the bags were centrifuged at 1000×*g* for 2 h and then cultured for 7 days. Cell number (F) and cell viability (G) were measured pre-centrifugation, post-centrifugation and after 4 and 7 days of culture. **Figure S2. Functional characteristics of CAR T-cells are not affected by spinoculation.** Flow cytometry data from killing assay experiments at 24 (A) and 48 h (B). C) CD19/22 Healthy Donor TNFα ELISA’s measuring cytokine levels in T-cell supernatant. D).CD19/22 Patient TNFα ELISA’s measuring cytokine levels in T-cell supernatant. E) CD22 TNFα ELISA’s measuring cytokine levels in T-cell supernatant. Mean and SD of triplicate wells are shown. *Indicates p ≤ 0.05, ****indicates p ≤ 0.0001 between the static and Sepax groups.

## Data Availability

The data analyzed are available from the corresponding author on reasonable request.
