# Supplementary material for: High efficiency closed-system gene transfer using automated spinoculation
Source: J Transl Med. 2021 Nov 24;19:474. doi: 10.1186/s12967-021-03126-4 (PMC8675485; doi:10.1186/s12967-021-03126-4)

Supplemental Figure 1: Retroviral gene transfer is enhanced by bag spinoculation

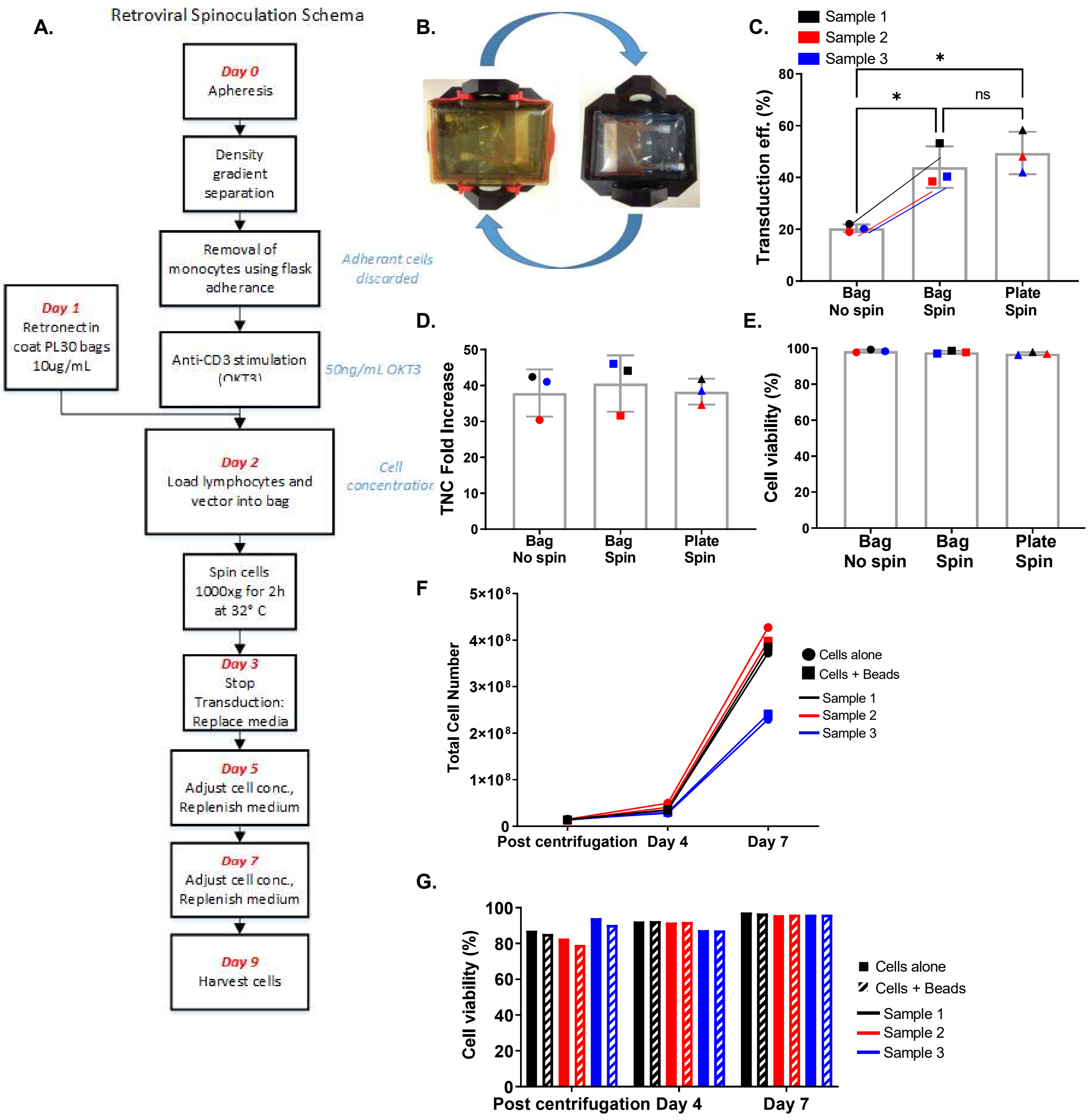

Supplemental Figure 2: Functional characteristics of CAR T-cells are not affected by spinoculation

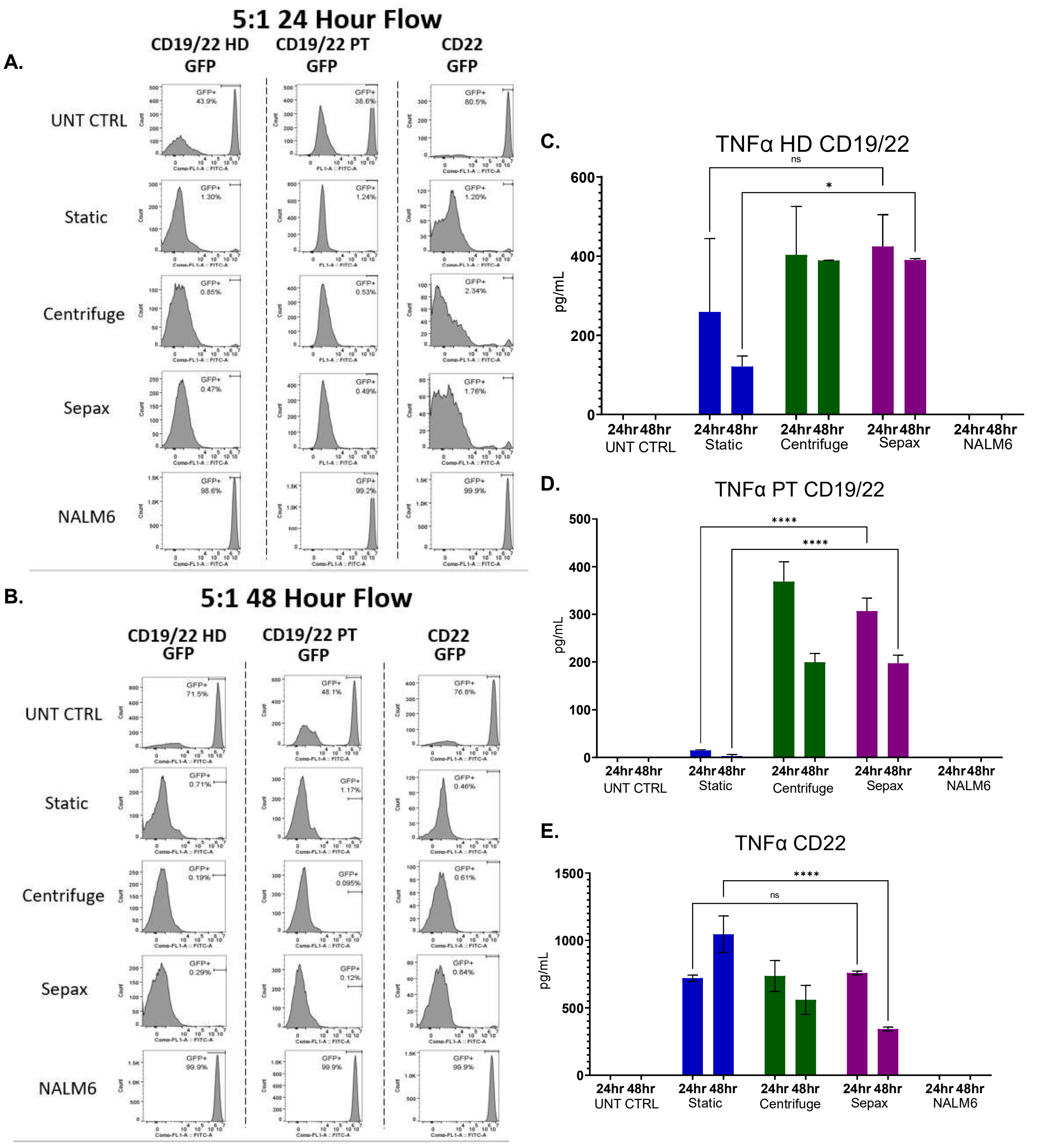

Supplement: Supplementary file 1 — Additional file 1: Figure S1. T-Cell Retroviral gene transfer is enhanced using a closed-system spinoculation with CD3/CD28 Dynabeads. Schema of culture (A1) and image of centrifuge bucket orientation (B). Lymphocytes were stimulated with rIL-2 and OKT3. After 2 days in culture cell were transduced with E6 TCR retroviral vector in 6 well plates using spinoculation or bags with and without spinoculation. The transduction process was repeated on day 3. After 7 days of culture the cells were evaluated for transduction efficiency (C), fold T cell expansion (D), and cell viability (E). PBMCs enriched for lymphocyte by density gradient centrifugation were placed in PL30 bags with rIL3 and OKT3 antibody with and without CD3/CD28 Dynabeads. To simulate spinoculation, the bags were centrifuged at 1000×g for 2 h and then cultured for 7 days. Cell number (F) and cell viability (G) were measured pre-centrifugation, post-centrifugation and after 4 and 7 days of culture. Figure S2. Functional characteristics of CAR T-cells are not affected by spinoculation. Flow cytometry data from killing assay experiments at 24 (A) and 48 h (B). C) CD19/22 Healthy Donor TNFα ELISA’s measuring cytokine levels in T-cell supernatant. D).CD19/22 Patient TNFα ELISA’s measuring cytokine levels in T-cell supernatant. E) CD22 TNFα ELISA’s measuring cytokine levels in T-cell supernatant. Mean and SD of triplicate wells are shown. *Indicates p ≤ 0.05, ****indicates p ≤ 0.0001 between the static and Sepax groups. [file 12967_2021_3126_MOESM1_ESM.pdf]
